# Dynamic Evolution of Cardiac Function and Glucose and Lipid Metabolism in Ovariectomized Rats and the Intervention Effect of Erxian Decoction

**DOI:** 10.1155/2022/8090868

**Published:** 2022-12-17

**Authors:** Yuhan Wang, Yanhua Jiang, Jing Hu, Ying Yang, Yanjun Liu, Haixia Liu, Zhiguo Zhang, Yanjing Chen

**Affiliations:** ^1^Institute of Basic Theory for Chinese Medicine, Chinese Academy of Chinese Medical Sciences, Beijing, China; ^2^Institute of Basic Research in Clinical Medicine, Chinese Academy of Chinese Medical Sciences, Beijing, China

## Abstract

**Aims:**

Abnormal changes in cardiac function have been reported in menopausal women, but there are few clinical studies on this topic. Erxian decoction (EXD) is a classic prescription that is widely used in the treatment of female menopausal diseases. The purpose of this study was to investigate the dynamic evolution of cardiac function and glucose and lipid metabolism in ovariectomized (OVX) rats and the intervention effect of EXD.

**Materials and Methods:**

The OVX climacteric rat model was established by bilateral ovariectomy. After successful modeling, the rats were randomly divided into four groups: the sham operation (SHAM) group (equal volumes of purified water), OVX group (equal volumes of purified water), estradiol (E_2_) group (1.8 × 10^−4^ g/kg), and EXD group (9 g/kg). Each group of rats was treated for 16 weeks. At the 4^th^, 8^th^, 12^th^, and 16^th^ weeks after treatment, the cardiac function of the rats in each group was evaluated by ultrasound. The coaxial method was used to measure blood pressure (BP). Serum endothelin-1 (ET-1) and angiotensin-2 (Ang II) levels were determined by the enzyme-linked immunosorbent assay (ELISA). The strip method was used to measure fasting blood glucose (FBG). The serum total cholesterol (TC) and triglyceride (TG) levels of rats were measured with the oxidase method. Direct methods were used to measure serum high-density lipoprotein (HDL-C) and low-density lipoprotein (LDL-C) levels. At week 16 of dosing, serum E_2_ levels were determined by E_2_ radioimmunoassay. The myocardium and uterus of the rats in each group were stained with HE (hematoxylin-eosin). The ultrastructure of the rat myocardium was observed by electron microscopy.

**Results:**

After the 16^th^ week of treatment, the serum E_2_ level decreased (*P* < 0.05), and the uterus was atrophied in OVX rats. The cardiac ejection fraction (EF%) decreased at 4 weeks after treatment, and systolic and diastolic function decreased after 12 weeks. After the 16^th^ week, the EF%, which reflects the “pump” function of the heart, decreased significantly (*P* < 0.05 or *P* < 0.01). At the 4^th^, 8^th^, 12^th^, and 16^th^ weeks of treatment, the systolic blood pressure (SBP), diastolic blood pressure (DBP), and mean pressure (MBP) of the rats in the OVX group increased with time (*P* < 0.05 or *P* < 0.01). The serum ET-1 and Ang II levels of rats in the OVX group increased (*P* < 0.05 or *P* < 0.01). In the OVX group, FBG was increased (*P* < 0.05 or *P* < 0.01), and blood lipids, especially LDL-C, were significantly increased (*P* < 0.05 or *P* < 0.01). After the 16^th^ week of treatment, the myocardial tissue of OVX rats showed obvious pathological changes. EXD significantly increased serum E_2_ levels (*P* < 0.01), decreased ET-1 and Ang II levels (*P* < 0.01), reduced the cardiac function risk factors BP, FBG, and blood lipids, and significantly improved cardiac function and structural changes in OVX rats (*P* < 0.05 or *P* < 0.01).

**Conclusions:**

EXD can improve abnormal cardiac structure and function in OVX rats.

## 1. Introduction

Globally, cardiovascular disease (CVD) is the most common cause of death, accounting for 31% of all deaths worldwide [1]. In addition to causing deaths, CVD places a large economic burden on society, accounting for a considerable proportion of healthcare expenditures and lost productivity worldwide [2]. Although CVD mortality has declined significantly over the past 40 years, CVD remains the leading cause of death among women [3]. Researchers have noted a link between E_2_ and CVD [3]. After menopause, risk factors for CVD are greatly increased in women due to the decrease in E_2_ levels [4]. Menopause-induced E_2_ reduction combined with aging can lead to unique female cardiac dysfunction [5, 6]. It has been reported that more menopausal women than men have heart failure (HF) with preserved EF%, that is, a left ventricle ejection fraction (LVEF) greater than 50% [5, 6]. A recent cross-sectional study showed that postmenopausal women undergo a series of pathological changes, such as inflammatory responses and vasodilatory dysfunction, due to E_2_ deficiency that leads to a decrease in myocardial contractile function [7]. However, it has also been reported that the decline in E_2_ levels in women in early menopause (within 1 year) actually increases LVEF and decreases diastolic function [8]. Specifically, in a certain period of menopause, the cardiac function status of women may vary with the time of menopause. However, the current literature that reports on abnormal cardiac function in menopausal women is inconsistent. Therefore, we speculate that women experience fluctuations in E_2_ changes during menopause with pathological dynamic changes in their cardiovascular system and cardiac function. In addition, there are few basic studies on cardiac function in menopausal model animals. Therefore, it is of great significance to explore the dynamic evolution of cardiac function in animal models of menopause.

Metabolic abnormalities are also typical symptoms of menopause [9, 10]. Blood lipid profiles, changes in blood glucose [9], and elevated BP levels [10] are common metabolic abnormalities in menopausal women, all of which are related to abnormal cardiac function [11]. All common pathological processes that affect left ventricular structure or function, such as hypertension [12], diabetes, and obesity, will affect left ventricular diastolic function [5]. However, the conclusions of clinical and basic research are not consistent. In a study of 350 perimenopausal women, abnormal HDL-C levels accounted for more than 50% of metabolic abnormalities, and high TG levels accounted for up to 40% of these abnormalities [13]. Compared with those in premenopausal women, both TG and TC levels were elevated in menopausal women [13]. A meta-analysis also showed that there was no significant difference in HDL-C levels in premenopausal and postmenopausal women, but TG, TC, and LDL-C levels were significantly higher in postmenopausal women than in premenopausal women [14]. A study on lipid levels in perimenopausal and postmenopausal women in China showed that postmenopausal women had lower HDL-C levels and higher TC, TG, and LDL-C levels than perimenopausal women [15]. Studies on the dynamic evolution of blood glucose and blood lipid profiles during menopause in OVX rats are rare. Therefore, we need to study the evolution of cardiac function in OVX rats and the dynamic evolution of its risk factors—BP, blood glucose, and blood lipids.

Hormone replacement therapy (HRT) was developed in response to the series of pathological changes caused by hormonal changes in menopausal women [16]. In basic research, E_2_ supplementation can also reduce pathological myocardial hypertrophy [16]. However, HRT has had mixed results. There is clinical evidence that exogenous E_2_ may reverse or delay menopause-related diastolic dysfunction and improve indicators of cardiac diastolic function [17]. However, primary and secondary clinical trials suggest that HRT increases the early risk of coronary heart disease [18]. In addition, some researchers have noticed that HRT in women can reduce the risk of CVD in the first year after menopause and that HRT after many years of menopause is ineffective or even harmful [19].

Phytoestrogens are found in a variety of herbs and are considered an alternative to HRT due to their structural similarity to E_2_ [20]. Erxian decoction (EXD) is a classic prescription that is widely used in the clinic for the treatment of menopausal syndrome [21]. EXD consists of six commonly used herbs, namely, *Curculigo orchioides Gaertn* (Xianmao), *Epimedii Folium* (Yinyanghuo), *Morinda officinalis Radix* (Bajitian), *Angelicae Sinensis Radix* (Danggui), *Phellodendri Chinensis Cortex* (Huangbo), and *Anemarrhenae Rhizoma* (Zhimu) [22]. Some studies on OVX rats show that EXD has the effect of increasing serum E_2_ [22, 23]. One research group assessing the effect of EXD on the cardiovascular system of OVX rats in the early stage found that the cardiac electrical activity of female OVX rats was abnormal, the electrophysiological properties of the myocardium were remodeled, the myocardium also had tissue structure remodeling, and myosin and other genes were also changed [24]. EXD can improve abnormal cardiac electrical activity in OVX rats, can regulate the expression of genes related to myocardial contraction, and has a protective effect on myocardial injury in OVX rats [24]. Therefore, this study used OVX rats as the model to discuss the dynamic evolution of cardiac function, BP and its traditional risk factors, blood glucose, and blood lipids in rats after ovariectomy. The function and effect of EXD interventions on glucose and lipid metabolism were also observed.

## 2. Materials and Methods

### 2.1. Drug Preparation

*Curculigo orchioides Gaertn* (Xianmao), *Epimedii Folium* (Yinyanghuo), *Morinda officinalis Radix* (Bajitian), *Angelicae Sinensis Radix* (Danggui), *Phellodendri Chinensis Cortex* (Huangbo), and *Anemarrhenae Rhizoma* (Zhimu) were purchased from Beijing Qiancao Traditional Chinese Medicine Co. Ltd.. We decocted the ingredients of EXD according to a mass ratio of 2 : 2 : 2 : 2 : 1 : 1. We added 10× and 8× (v/w) distilled water for the first and second times, respectively. It was heated by a slow fire until boiling and maintained for 1 hour after boiling. The medicinal solution was mixed and concentrated to 0.9 g (raw herbs)/mL after mixing twice and stored in a refrigerator at 4°C until use. For estradiol valerate tablets (Bayer Health Care Co. Ltd., Guangzhou Branch, batch number: 398A), 9.2 mg was dissolved in 500 mL of normal saline to prepare liquid medicine with a concentration of 0.018 g/L and stored in a refrigerator at 4°C until use.

### 2.2. Experimental Animals

In this study, 32 female SPF grade Sprague-Dawley rats (average weight of 230 ± 15.0 g; 10 weeks of age) were obtained from SPF (Beijing) Biotechnology Co. Ltd. (SCXK (JING) 2016-0002). The animals were housed in a clean animal room at the Laboratory Animal Center of the Institute of Basic Theories of Chinese Medicine, China Academy of Chinese Medical Sciences. They were housed under standard conditions of temperature (24 ± 2°C) and humidity (45∼60%) under a 12 : 12-h day/night cycle. Food and water were provided ad libitum. This protocol was authorized by the Institutional Ethics Committee of the China Academy of Chinese Medical Sciences (approval number: 2018-058). After 1 week of adaptive feeding, rats in the modeling operation group (*n* = 24) were anesthetized by intraperitoneal injection of 3% sodium pentobarbital (1.3 mL/kg), and the ovaries were removed. In the SHAM group (*n* = 8), small pieces of adipose tissue on both sides of the abdominal cavity were removed, and the ovaries were preserved. The rats were intraperitoneally injected with penicillin sodium 240,000 U/kg/d for 3 consecutive days after the operation to prevent infection. One week after the operation, the rats were tested by the vaginal smear for 5 consecutive days. The estrous cycle remained intact for the rats in the SHAM group but had disappeared in the OVX group. Vaginal smears were used to confirm the success of the model [23]. The successfully modeled rats were randomly divided into the model group (OVX group, *n* = 8), E_2_ group (*n* = 8), and EXD group (*n* = 8). Two weeks after ovariectomy, the rats were intragastrically administered their treatment once a day. It was stopped for 6 days for 1 consecutive day for a total of 16 weeks. The doses of each group were as follows: EXD group, 9 g/kg; E_2_ group, 1.8 × 10^−4^ g/kg; and SHAM group and OVX group, equal volumes of purified water. The mental state, posture, fur color, mobility, eye cleft mucosa color, auricle color, and feces and other general conditions of rats were carefully observed every day. The rats in each group were weighed and recorded at a fixed time each week.

### 2.3. Detection of Cardiac Function

After the 4^th^, 8^th^, 12^th^, and 16^th^ weeks of treatment, 6 rats in each group were fasted for 12 hours. Echocardiography (UCG) was performed using an ultrahigh resolution small animal ultrasound imaging system (VisualSonics, Canada, Vevo2100) to assess cardiac structure and cardiac function. After the depilatory cream (Nair Australia, LL6084) was applied to the clavicle and the xiphoid process of rats, rats were laid in a supine position and fixed on the operating table. Then, the coupling agent was applied. The rats were anesthetized by inhalation of a mixture of isoflurane and pure oxygen using a ventilator inhalation anesthesia machine (VisualSonics, Canada, VS4083), and the heart rate of the rats was maintained at approximately 350 beats/min. Then, we used a Vevo2100 ultra-high-resolution small-animal ultrasound imaging system and the MS-250 21 Hz probe to obtain the short axis view of the left ventricle of the rats and made the M-type UCG sampling line at the level of the papillary muscle. At least 10 cardiac cycles were continuously recorded, and the following parameters were measured and calculated: EF%, left ventricular short axis shortening rate (FS%), left ventricular end systolic diameter (LVIDs), left ventricular posterior wall end systolic thickness (LVPWs), left ventricular end systolic volume (LVVols), left ventricular end diastolic diameter (LVIDd), left ventricular posterior wall end diastolic thickness (LVPWd), and left ventricular end diastolic volume (LVVold). All indicators were the average of 3 cardiac cycle parameters.

### 2.4. Detection of BP and Heart Rate (HR)

After the 4^th^, 8^th^, 12^th^, and 16^th^ weeks of treatment, a noninvasive BP meter for rats (Japan Softron Corporation, BP-98A) was used to measure the tail artery SBP, DBP, MBP, and HR of the rats in each group using the coaxial method. During the experiment, rats were allowed to adapt to the dark experimental environment in advance, and the insulation cylinder was kept at 38°C. After rats were in a stable state, BP and HR were measured. Each rat was continuously measured 3 times, and each data point was the average of 3 measurements. A BP-98A noninvasive BP acquisition system was used to collect data.

### 2.5. Biochemical Analysis

After the 4^th^, 8^th^, 12^th^, and 16^th^ weeks of treatment, each group of rats fasted for 12 hours in advance. FBG was measured using a blood glucose meter and test strips (USA Johnson & Johnson). Then, blood samples (0.2 mL) were collected by inserting capillary tubes into the periorbital plexus. After the 16^th^ week of treatment, the rats in each group were anesthetized with an intraperitoneal injection of 3% sodium pentobarbital (1.3 mL/kg) after 12 h of fasting, and abdominal aortic blood was collected. Serum was isolated by centrifugation (4°C, 5000 rpm for 15 min) using a low-temperature centrifuge (American Thermo Company, Multifuge X1R) and stored at −80°C until use. Serum TC and TG levels in rats were measured by the oxidative enzyme method (Intech Innovation (Xiamen) Technology Co. Ltd, batch numbers: 671030E12, 671031E12). Serum HDL-C and LDL-C levels were measured by a direct method ((Intech Innovation (Xiamen) Technology Co., Ltd, batch numbers: 671016E12, 671024E12)). Serum ET-1 and Ang II contents were detected by using ELISA kits (Nanjing Jiancheng Institute of Biological Engineering, batch numbers: 20181121, 20181214), and an automatic microplate reader (American Thermo Company, Multiskan EX Primary EIA V. 2.3) was used to measure absorbance *A*. After the 16^th^ week of treatment, serum E_2_ levels were detected by using an E_2_ radioimmunoassay kit (Beijing North Institute of Biotechnology Co., batch number: 20190320) and using a gamma radioimmunoassay counter (Xi'an Nuclear Instrument Factory, XH-6080*γ*).

### 2.6. Histological Staining

After the 16^th^ week of treatment, rats were anesthetized by an intraperitoneal injection of 3% sodium pentobarbital (1.3 mL/kg). The rat heart cross section was obtained. It was immediately rinsed with ice-cold saline and then fixed in 4% paraformaldehyde solution (Servicebio, China, batch number: ZH193506) for subsequent HE staining (Beijing Jinqiao Yatu Biotechnology Co. Ltd., batch numbers: 180301, 180601) of ventricular tissue. The myocardium sample (1 mm × 1 mm × 1 mm) was cut longitudinally from the left ventricular muscle. It was immediately placed in 4% glutaraldehyde (Servicebio, China, batch number: ZH193506) solution for fixation and stored at 4°C for testing. Subsequently, we incised ipsilateral uterine tissue approximately 1 cm above the Y-shaped uterine junction, immediately rinsed it with ice-cold saline, and immediately placed it in 4% paraformaldehyde solution for subsequent HE staining. The above samples were stored at 4°C until testing. Both HE and electron microscopy staining were performed according to the manufacturer's instructions.

### 2.7. Statistical Analysis

SPSS 20.0 statistical software was used for analysis, and the data are expressed as the mean ± standard deviation. One-way ANOVA was used for comparisons between multiple groups, and the LSD test was used for pairwise comparisons when the variance was homogeneous. The Kruskal–Wallis nonparametric test was used when the variance was not homogeneous, and the Bonferroni method was used for pairwise comparisons. A *P* value <0.05 was considered statistically significant.

## 3. Results

### 3.1. Effect of EXD on Body Weight and Uterine Coefficient in OVX Rats after the 16^th^ Week of Treatment

**Figure 1:**
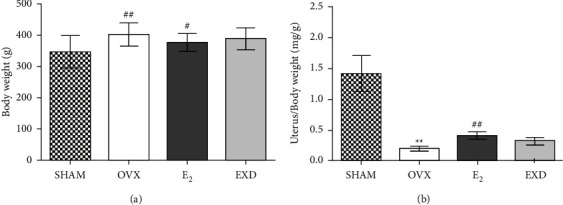
Effect of EXD on body weight (a) and uterine coefficient (b) after the 16^th^ week of treatment. Data are presented as the mean ± SD. ^*∗*^*P* < 0.05, ^*∗∗*^*P* < 0.01 vs. SHAM group; ^#^*P* < 0.05, ^##^*P* < 0.01 vs. OVX group. *n* = 8 per group.

Compared with that in the SHAM group, the body weight of the rats in the OVX group increased significantly, and the uterine coefficient decreased significantly (*P* < 0.01). Compared with that in the OVX group, the body weight of the rats in the E_2_ group decreased (*P* < 0.05), and the uterine coefficient increased significantly (*P* < 0.01). However, there was no significant difference in the body weight or uterine coefficient of the rats in the EXD group. Body weight and uterine coefficient graphs are shown in Figures 1(a) and 1(b).

### 3.2. Effect of EXD on Pathological Changes in the Uterus after the 16^th^ Week of Treatment

**Figure 2:**
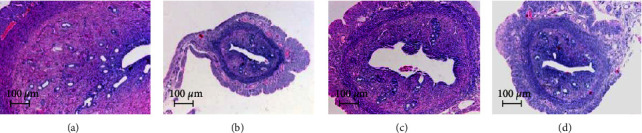
Effect of EXD on pathological changes in the uterus after the 16^th^ week of treatment (HE staining, 50×). (a) SHAM group; (b) OVX group; (c) E_2_group; (d) EXD group.

After the 16^th^ week of treatment, the morphological changes in the uteri of the rats in each group were observed under a light microscope. The endometrium of the rats in the SHAM group was thicker, with abundant glands. The monolayer columnar epithelium was neatly arranged (Figure 2(a)). In the OVX group, uterine atrophy occurred, the wall of the tube became significantly thinner, and the lumen narrowed (Figure 2(b)). Compared with the OVX group, the endometrium in the E_2_ group was covered by a single layer of cuboidal epithelium. In addition, the single layer of columnar epithelium was irregularly arranged, and the number of endometrial glands was higher (Figure 2(c)). The endometrium of rats in the EXD group was covered with a single layer of squamous epithelium or a single layer of cuboidal epithelium. It had fewer endometrial glands and occasional secretions (Figure 2(d)).

### 3.3. Effect of EXD on Serum E_2_ Content after the 16^th^ Week of Treatment

**Figure 3:**
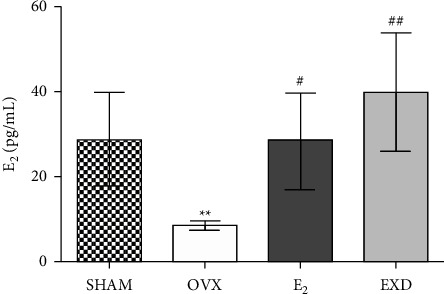
Effect of EXD on serum E_2_ content after the 16^th^ week of treatment. Data are presented as the mean ± SD. ^*∗*^*P* < 0.05, ^*∗∗*^*P* < 0.01 vs. SHAM group; ^#^*P* < 0.05, ^##^*P* < 0.01 vs. OVX group. *n* = 8 per group.

Compared with that in the SHAM group, after 16 weeks of treatment, the serum E_2_ content of the rats in the OVX group decreased (*P* < 0.05). In addition, compared with that in the OVX group, the serum E_2_ content of the rats in the E_2_ group increased (*P* < 0.05). Furthermore, the serum E_2_ content of the rats in the EXD group increased significantly (*P* < 0.01). The above E_2_ results are shown in Figure 3.

### 3.4. Effect of EXD on Pathological Changes in the Left Ventricular Myocardium after the 16^th^ Week of Treatment

**Figure 4:**
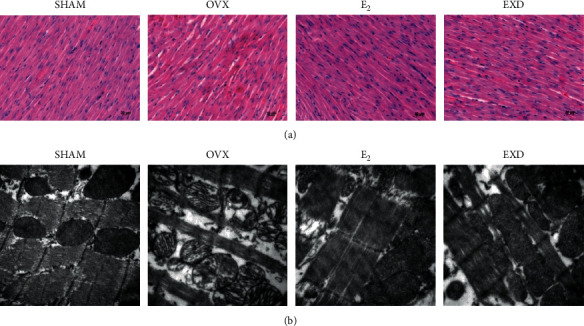
Effect of EXD on pathological changes in the left ventricular myocardium after the 16^th^ week of treatment. (a) Pathological changes in the left ventricular myocardium after the 16^th^ week of administration (HE staining, 200×). (b) Ultrastructure of the left ventricular myocardium after the 16^th^ week of administration (electron microscope, 30000×).

After the 16^th^ week of treatment, the results of myocardial HE staining and myocardial transmission electron microscopy showed that in the SHAM group, myocardial stripes were arranged regularly and clearly, myocardial cells were arranged neatly and densely, myocardial cell filaments were neat, and sarcomeres were regular and clear. The myocardial mitochondrial membrane was intact, and the mitochondrial crista structure was clear in the SHAM group. Compared with the SHAM group, the myocardial tissue of the OVX group showed atrophy and thinning of myocardial fibers, enlarged myocardial cell space, obvious focal eosinophilic changes in myocardial cells, and deep staining of the nuclei. The ultrastructure in the OVX group showed that the arrangement of myocardial myofibrils was disordered, the arrangement of myofilaments was not neat, the structure of sarcomeres was blurred, the nuclei of myocardial cells were pyknotic, and the cristae of myocardial mitochondria appeared obviously broken and sparse. Compared with those in the OVX group, the rats in the E_2_ group had obvious myocardial striations, occasional atrophy of myocardial cells, focal interstitial hyperplasia, a neat arrangement of myofilaments, a clear mitochondrial crista structure, and no obvious swelling. Atrophy and stromal hyperplasia of myocardial cells in the EXD group were lighter, the mitochondrial membrane was intact, and the mitochondrial crista structure was clearer than that in the OVX group. The above results indicate that both E_2_ and EXD can improve the pathological changes in myocardial structure in OVX rats. The above pathological findings are shown in Figures 4(a) and 4(b).

### 3.5. Effect of EXD on Cardiac Function after the 4^th^, 8^th^, 12^th^, and 16^th^ Week of Treatment

**Figure 5:**
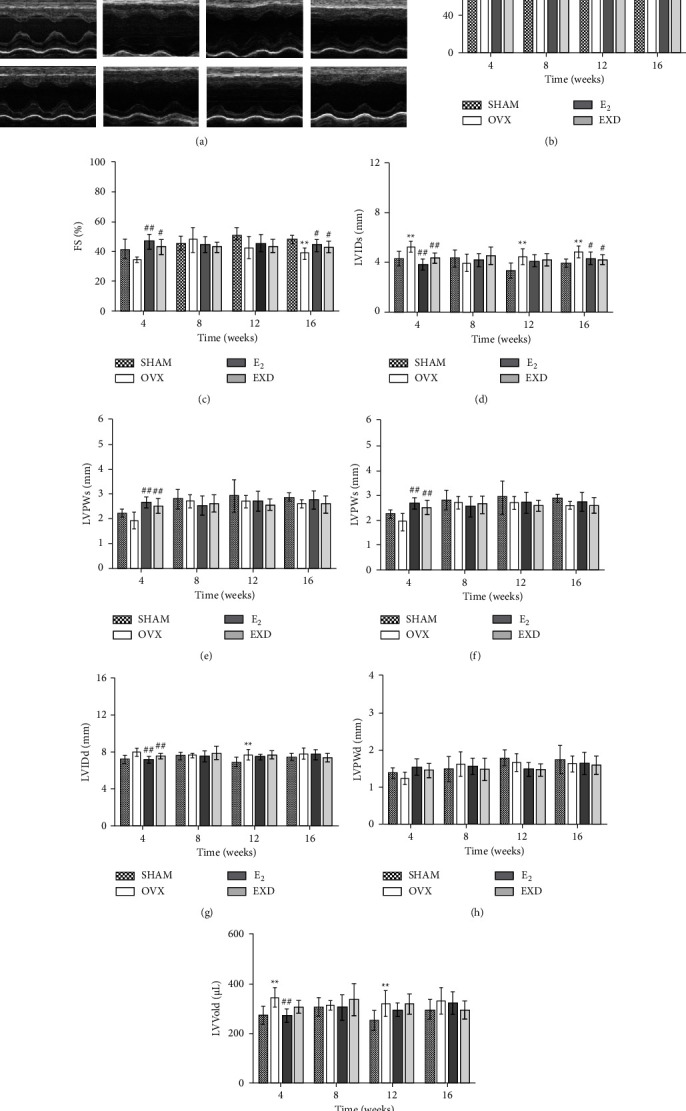
Effect of EXD on cardiac function after the 4^th^, 8^th^, 12^th^, and 16^th^ week of treatment. (a) Representative echocardiography pictures of M mode images. (b) Ejection fraction (EF%). (c) Fractional shortening (FS%). (d): Left ventricular (LV) end systolic inner diameter (LVIDs). (e) LV posterior wall thickness in end systole (LVPWs). (f) LV end systolic volume (LVVols). (g) LV end diastolic inner diameter (LVIDd). (h): LV posterior wall thickness in end diastole (LVWPd). (i) LV end diastolic volume (LVVols). Data are presented as the mean ± SD. ^*∗*^*P* < 0.05, ^*∗∗*^*P* < 0.01 vs. SHAM group; ^#^*P* < 0.05, ^##^*P* < 0.01 vs. OVX group. *n* = 6 per group.

Compared with that in the SHAM group, after the 4^th^ week of treatment, EF% of rats in the OVX group decreased (*P* < 0.05), and LVIDs, LVVols, and LVVold were significantly increased (*P* < 0.01). After the 8^th^ week of treatment, there were no significant differences in various indicators of cardiac function in the OVX group. In the OVX group, LVIDs, LVVols, LVIDd, and LVVold were significantly increased after the 12^th^ week of treatment (*P* < 0.01). At the 16^th^ week of treatment, both EF% and FS% were significantly decreased in the model group (*P* < 0.01), and LVIDs and LVVols increased significantly (*P* < 0.01). Compared with those in the OVX group, after the 4^th^ week of treatment, EF%, FS%, and LVPWs of the E_2_ group were significantly increased (*P* < 0.01), and the LVIDs, LVVols, LVIDd, and LVVold were significantly decreased (*P* < 0.01). EF%, FS%, and LVPWs of the EXD group were increased to different degrees (*P* < 0.05 or *P* < 0.01), and LVIDs, LVVols, and LVIDd were decreased to different degrees (*P* < 0.05 or *P* < 0.01). After the 12^th^ week of treatment, the systolic and diastolic function indices of the rats in the E_2_ group and the EXD group showed a trend toward improvement, but there was no significant difference. After the 16^th^ week of treatment, EF% and FS% of the E_2_ group and EXD group were significantly increased (*P* < 0.01), LVIDs was significantly decreased (*P* < 0.01), and LVVols was decreased (*P* < 0.05). The above pathological findings are shown in Figures 5(a) and 5(i).

### 3.6. Effect of EXD on BP and HR after the 4^th^, 8^th^, 12^th^, and 16^th^ Week of Treatment

**Figure 6:**
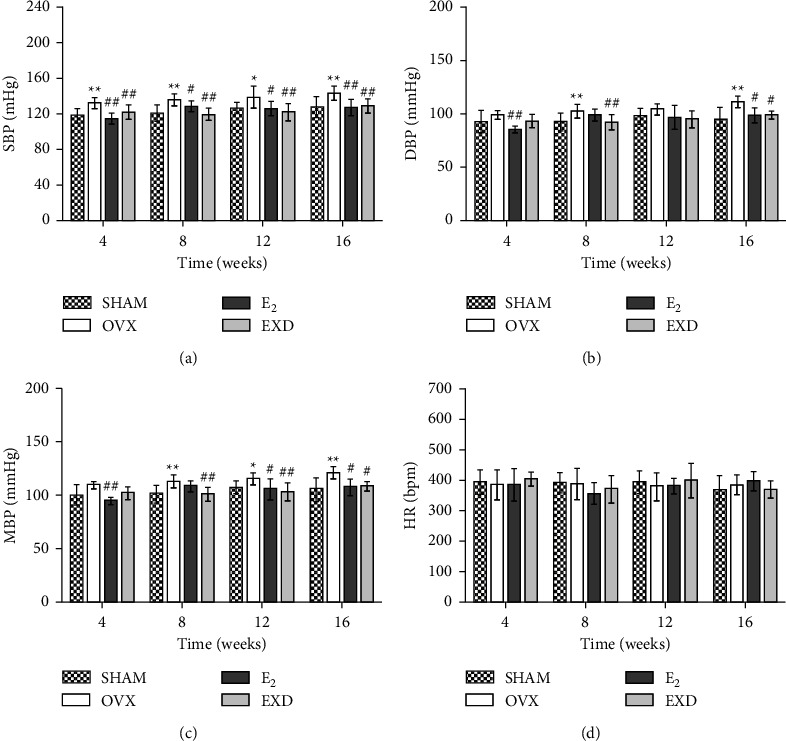
Effect of EXD on blood pressure (BP) and the heart rate (HR) after the 4^th^, 8^th^, 12^th^, and 16^th^ week of treatment. (a) Systolic blood pressure (SBP). (b) Diastolic blood pressure (DBP). (c) Mean pressure (MBP). (d) Heart rate (HR). Data are presented as the mean ± SD. ^*∗*^*P* < 0.05, ^*∗∗*^*P* < 0.01 vs. SHAM group; ^#^*P* < 0.05, ^##^*P* < 0.01 vs. OVX group. *n* = 8 per group.

Compared with those in the SHAM group, after the 4^th^, 8^th^, 12^th^, and 16^th^ week of treatment, the SBP, DBP, and MBP were increased in the OVX group (*P* < 0.05 or *P* < 0.01), and this increase was gradual. There was no significant difference in HR in the OVX group. Compared with those in the OVX group, after the 4^th^, 8^th^, 12^th^, and 16^th^ week of treatment, the SBP, DBP, and MBP were decreased in the E_2_ group (*P* < 0.05 or *P* < 0.01). In the EXD group, SBP decreased significantly (*P* < 0.01), and both DBP and MBP were decreased (*P* < 0.05 or *P* < 0.01). The above results indicate that EXD reduces the increased SBP mainly in OVX rats, and this effect is better than that of E_2_. The above results are shown in Figures 6(a)∼6(d).

### 3.7. Effect of EXD on Serum ET-1 and Ang II Levels after the 4^th^, 8^th^, 12^th^, and 16^th^ Week of Treatment

**Figure 7:**
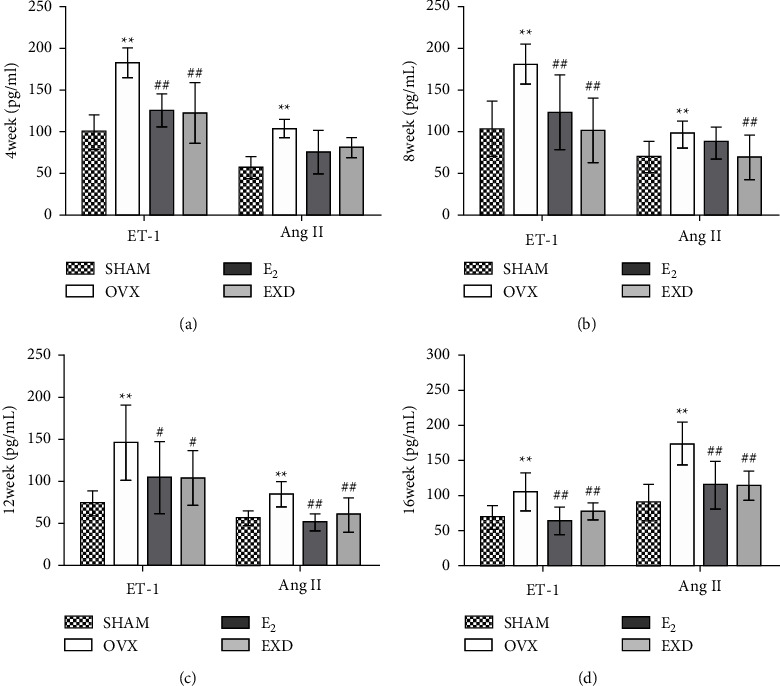
Effect of EXD on serum ET-1 and Ang II levels after the (a) 4^th^, (b) 8^th^, (c) 12^th^, and (d) 16^th^ week of treatment. Data are presented as the mean ± SD. ^*∗*^*P* < 0.05, ^*∗∗*^*P* < 0.01 vs. SHAM group; ^#^*P* < 0.05, ^##^*P* < 0.01 vs. OVX group. *n* = 8 per group.

Compared with those in the SHAM group, after the 4^th^, 8^th^, 12^th^, and 16^th^ week of treatment, the serum levels of ET-1 and Ang II in the OVX group were significantly increased (*P* < 0.01). Compared with those in the OVX group, the serum ET-1 levels in the E_2_ group and the EXD group were significantly decreased (*P* < 0.05 or *P* < 0.01). The serum Ang II levels in the E_2_ group and the EXD group were significantly decreased at the 8^th^, 12^th^, and 16^th^ week of treatment (*P* < 0.01). The above results show that both E_2_ and EXD can reduce the serum levels of ET-1 and Ang II in OVX rats and that the effects of the two are equivalent. The above results are shown in Figures 7(a)∼7(d).

### 3.8. Effect of EXD on FBG after the 4^th^, 8^th^, 12^th^, and 16^th^ Week of Treatment

**Figure 8:**
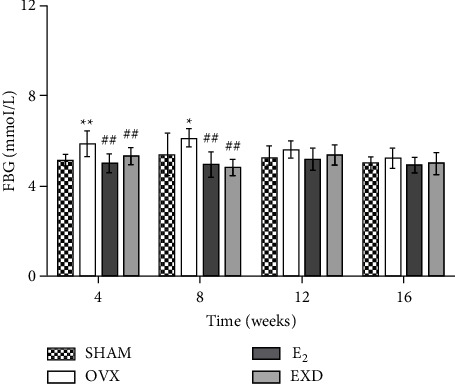
Effect of EXD on fasting blood glucose (FBG) after the 4^th^, 8^th^, 12^th^, and 16^th^ week of treatment. Data are presented as the mean ± SD. ^*∗*^*P* < 0.05, ^*∗∗*^*P* < 0.01 vs. SHAM group; ^#^*P* < 0.05, ^##^*P* < 0.01 vs. OVX group. *n* = 8 per group.

Compared with that in the SHAM group, FBG was significantly increased in the OVX group after the 4^th^ week of treatment (*P* < 0.01) and after the 8^th^ week of treatment (*P* < 0.05). In addition, there was a trend toward an increase in FBG in the OVX group after the 12^th^ and 16^th^ weeks of treatment, but the change was not significant. Compared with that in the OVX group, the FBG of rats in the E_2_ and OVX groups decreased significantly after the 4^th^ and 8^th^ week of treatment (*P* < 0.01). The above results show that E_2_ and EXD can reduce the FBG levels of OVX rats after the 4^th^ and 8^th^ week of treatment. There was no significant difference in the FBG levels of the rats in each group after the 12^th^ and 16^th^ week of treatment. The above results are shown in Figure 8.

### 3.9. Effect of EXD on Serum TC, TG, HDL-C, and LDL-C Levels at the 4^th^, 8^th^, 12^th^, and 16^th^ Week of Treatment

**Figure 9:**
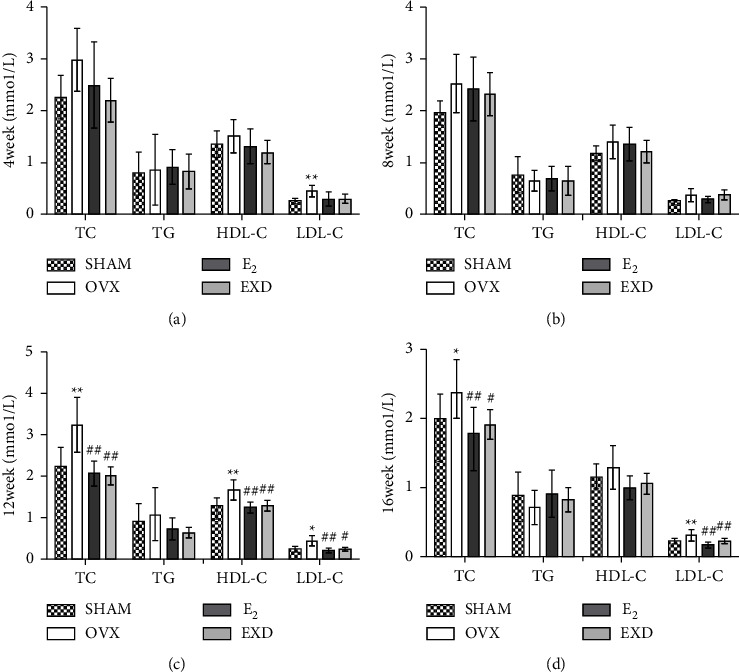
Effect of EXD on serum total cholesterol (TC), triglyceride (TG), high-density lipoprotein (HDL-C), and low-density lipoprotein (LDL-C) content after the (a) 4^th^, (b) 8^th^, (c) 12^th^, and (d) 16^th^ week of treatment. Data are presented as the mean ± SD. ^*∗*^*P* < 0.05, ^*∗∗*^*P* < 0.01 vs. SHAM group; ^#^*P* < 0.05, ^##^*P* < 0.01 vs. OVX group. *n* = 8 per group.

Compared with that in the SHAM group, after the 4^th^ week of treatment, the level of serum LDL-C in the OVX group increased significantly (*P* < 0.01). After the 8^th^ week of treatment, there was no significant difference in serum TC, TG, HDL-C, and LDL-C levels. After the 12^th^ week of treatment, the serum TC and HDL-C levels increased significantly (*P* < 0.01), as did serum LDL-C levels (*P* < 0.05). After the 16^th^ week of treatment, serum TC levels significantly increased (*P* < 0.05), as did serum LDL-C levels (*P* < 0.01). Compared with those in the OVX group, after the 4^th^ and 8^th^ week of treatment, there were no significant differences in the serum TC, TG, HDL-C, and LDL-C levels in the E_2_ group. After the 12^th^ week of treatment, the serum TC, HDL-C, and LDL-C levels were significantly reduced (*P* < 0.01). Furthermore, after the 16^th^ week of treatment, the serum TC and LDL-C levels were significantly reduced (*P* < 0.01). After the 4^th^ and 8^th^ week of treatment, there were no significant differences in the serum TC, TG, HDL-C, and LDL-C levels of the rats in the EXD group. After the 12^th^ week of treatment, the serum TC and HDL-C levels were significantly reduced (*P* < 0.01), as were serum LDL-C levels (*P* < 0.05). After the 16^th^ week of treatment, the serum TC level significantly decreased (*P* < 0.05), as did the serum LDL-C level (*P* < 0.01). The above results are shown in Figures 9(a)∼9(d).

## 4. Discussion

Clinically, cardiac function is closely related to HF. HF is an important cause of increased morbidity and mortality in patients [25]. The incidence increases rapidly with age [26]. HF is caused by abnormal diastolic ventricular filling and decreased systolic ejection [25]. The left ventricular HF can be divided into HF with a preserved ejection fraction (HfpEF) and HF with a reduced ejection fraction (HFrEF). HfpEF is defined as LVEF > 50%, and HFrEF is defined as LVEF  < 40% [25]. At present, research on HF and heart function in menopausal women has not yet reached a unified conclusion [6]. Many clinical studies have indicated that menopausal women are more likely to suffer from HfpEF than men, with women being almost twice as likely as men to suffer from it [6]. However, it has also been reported that after adjusting for age and risk factors, the prevalence in women and men does not differ significantly [27]. According to the study by Alexanderson-Rosas et al., cardiac systolic function is decreased in menopausal women [7]. There are also studies suggesting that LVEF increases in early menopausal women, while diastolic function decreases [8]. Basic research has also addressed the issue of abnormal cardiac function in OVX animal models, and it is believed that various cardiac function parameters of OVX rats have abnormal changes [27, 28].

In view of the adverse effects of estrogen on clinical studies, traditional Chinese medicine with estrogen-like effects has become a research hotspot [29, 30]. EXD is a classic prescription for the treatment of menopausal syndrome in females. It was developed by the modern and famous doctor Zhang Bone [21]. It can supplement serum E_2_ in OVX rats [22]. There are abundant E_2_ receptors in rodents, and the biological roles of E_2_ and E_2_ receptors involve their effects on genomic or nongenomic pathways or the direct activation of estrogen receptors by E_2_ in mitochondria [31–33].

In this study, we successfully established a rat model of menopause by bilateral ovariectomy [34]. OVX rats showed pathological changes manifesting as decreased E_2_ levels, increased body weight, a decreased uterine coefficient, and uterine atrophy due to ovariectomy [35]. EXD may play a role in reducing uterine atrophy by increasing serum E_2_ content in OVX rats, which is consistent with the findings of Vijayanarayana et al. [35]. Vijayanarayana et al. found that curcuma alcohol extract can promote endometrial hyperplasia [35]. They also found that it can significantly increase vaginal keratinization and uterine quality in rats, thereby exerting estrogen-like effects [35]. In addition, the EXD group rats showed an increase in the uterine coefficient, but the difference did not reach statistical significance. Icariin is one of the components of *Epimedii Folium* (Yinyanghuo) [36]. Icariin increases E_2_ and promotes the expression of estrogen receptors [36]. However, in this experiment, EXD had no obvious effect on body weight. Some researchers believe that body composition may play a more important role in cardiovascular disease and that increased lean mass in obese individuals may improve protection in patients with coronary heart disease without inflammation [37].

Ultrastructure showed that the arrangement of myofibrils in OVX rats was disordered, the sarcomere structure was blurred, the nuclei of the myocardium were pyknotic, and the mitochondrial cristae of the myocardium were obviously broken and sparse. The myocardial mitochondrial membrane of rats in the EXD group was intact, and the mitochondrial cristae were clear without swelling. These findings suggest that EXD can improve myocardial energy metabolism, mitochondrial structure, and mitochondrial function in OVX rats. E_2_ plays an active role in improving the mitochondrial function process [5]. Icariside II can reduce myocardial fibrosis in spontaneously hypertensive rats, inhibit cardiomyocyte apoptosis, reduce myocardial ROS production, and inhibit mitochondrial apoptosis through the ASK1-JNK/p38 signaling pathway [38]. The protective effect of EXD in OVX rats occurs through the above pharmacological effects.

The results of the cardiac function of rats at different times after treatment showed that after 4 weeks of treatment, cardiac systolic and diastolic function decreased in OVX rats. After 8 weeks of treatment, the parameters of cardiac function in OVX rats showed no obvious abnormalities. After 12 weeks of treatment, the cardiac systolic and diastolic functions of OVX rats decreased, and the cardiac systolic function of OVX rats decreased at 16 weeks. EXD can obviously improve the abovementioned changes in cardiac function. After comparing the cardiac function parameters of OVX rats, we believe that the decrease in cardiac function in rats after treatment for 4 weeks is due to surgical reasons and that the cardiac function of rats after treatment for 8 weeks is still in the compensation period. At the 12^th^ week, the systolic and diastolic functions of OVX rats were decreased. At that time, the OVX rats were at a low E_2_ level for a long period. To maintain normal blood perfusion, the myocardium showed compensatory changes in the prolonged ejection time and shortened ventricular diastolic time. This process further reduced ventricular diastolic blood perfusion, resulting in left ventricular compensation [29]. This compensation increases the inner diameter, and myocardial diastolic dysfunction occurs [29]. At 16 weeks of treatment, the increase in LVVold in OVX rats did not reach statistical significance, but the parameters EF% and FS%, reflecting cardiac pump function, decreased significantly. EXD can increase serum E_2_ levels in OVX rats, and E_2_ plays a positive role in improving myocardial relaxation, myocardial cell Ca^2+^ homeostasis, and antimyocardial fibrosis [5]. Pharmacological studies have shown that flavonoid icariside II, the main active component of *Epimedii Folium* (Yinyanghuo), can improve abnormal ventricular remodeling in spontaneously hypertensive rats [38]. Therefore, EXD may reverse the abnormal cardiac systolic and diastolic function of OVX rats in the abovementioned ways. It also protects cardiac function and improves myocardial pathological changes in OVX rats to a certain extent.

The incidence of hypertension is increased in postmenopausal women, and hypertension is also an important cause of cardiac left ventricular remodeling [39]. Clinically, the treatment of patients with HFpEF focuses on lowering BP [25]. ET-1 is an important player in the occurrence and development of hypertension [40]. The renin-angiotensin system (RAS) is an important regulator of renal function and arterial pressure [41]. Ang II can be activated and has a strong vasoconstrictive effect [42]. Ang II can also induce the production of ET, which further produces the effect of vasoconstriction [42]. Elevated BP has been demonstrated in OVX model animals [43], and E_2_ deficiency is closely related to the occurrence of hypertension [41, 44, 45]. The calcium channel antagonist action of E_2_ can inhibit ET-1, thereby dilating blood vessels [46]. E_2_ also reduces BP by inhibiting the RAS system [41]. In this experiment, after 16 weeks of treatment, the SBP of OVX rats was significantly increased, which may be related to the increased serum ET-1 and Ang II levels. EXD may play an important role in lowering BP by reducing serum ET-1 and Ang II levels in OVX rats. Another study showed that intragastric treatment with icariside II reduced blood pressure in spontaneously hypertensive rats [38]. Clinical studies have shown that EXD can further reduce SBP and DBP in postmenopausal female hypertensive patients on the basis of benazepril treatment and can also regulate E_2_ levels [47]. Therefore, EXD may play an important role in lowering BP through the above pharmacological effects.

Changes in blood lipid profiles and blood glucose levels are common metabolic diseases in menopausal women, and blood glucose and blood lipid abnormalities are related to abnormal cardiac function [11]. Glucose intolerance and insulin resistance have been reported to be associated with diastolic dysfunction even before the development of diabetes [48]. There is also downregulation of aortic estrogen receptor expression in patients with diabetes [5]. Lipotoxicity and glucotoxicity can also lead to the impairment of vascular function, cardiac contractility, and oxidative stress, among others [49]. Even in the absence of overt coronary artery disease, metabolic abnormalities can lead to cardiac remodeling and cardiac dysfunction [11]. Minta et al. reported that 6 weeks after oophorectomy, high-fat-diet-fed female rats can develop dysglycemia and dyslipidemia [33]. According to Delgobo et al., the E_2_ level will rapidly decrease in the short term in the OVX model without affecting oxidative damage, inflammation, or lipid metabolism. In OVX rats with long-term decreases in E_2_ levels, TG, glucose, and LDL-C levels are unchanged [50]. From the results of this study, it can be seen that an increase in serum TG levels was never evident in OVX rats. However, after 4 weeks of treatment, the LDL-C level increased significantly. After 12 weeks, the TC, HDL-C, and LDL-C levels of OVX rats increased significantly. After 16 weeks of treatment, the TC and LDL levels of OVX rats increased significantly. The blood glucose of OVX rats increased significantly at 4 weeks and 8 weeks after treatment, and there was no abnormal change at 12 or 16 weeks. Rats in the EXD group had pathological changes in blood lipids and decreased blood glucose levels at different stages. We speculated that the abnormal changes in the blood lipid profile of OVX rats after 4 weeks of treatment were due to their postoperative stimulation and that the blood lipids of OVX rats were in a compensatory phase after 8 weeks. One study showed that icariin reduced serum total TC, TG, and LDL levels and increased HDL levels in rats with nonalcoholic fatty liver disease [51]. EXD combined with Xiaochaihu decoction and metformin hydrochloride tablets can effectively improve FBG in patients with type 2 diabetes mellitus and menopausal syndrome [52]. EXD improves the changes in glucose and lipid metabolism in OVX rats at different stages, which may be related to the increase in serum E_2_ levels and the lipid-lowering effect of icariin on OVX rats.

## 5. Conclusions

In this study, OVX rats showed decreased cardiac systolic and diastolic functions at the 4^th^ week after treatment, and EF% and FS%, reflecting cardiac pump function, were significantly decreased at the 16^th^ week. The BP of OVX rats increased at the 4^th^ week after treatment, and SBP, DBP, and pulse pressure difference increased gradually with time. In OVX rats, FBG increased in the early stage after ovariectomy, and there was no significant difference in FBG levels between the groups in the later period. Serum levels of LDL-C in OVX rats were significantly increased at the 4^th^ week of treatment, and there was no significant difference in blood lipid levels between the groups at the 8^th^ week. Overall, serum levels of LDL-C were elevated in OVX rats throughout the experimental observation period. EXD can improve myocardial histological reconstruction, lower BP, regulate blood hormones and bioactive substances, lower LDL-C in blood, improve cardiac structure and function, and significantly improve glucose and lipid metabolism disorders in castrated rats. This study provided an experimental basis for an indepth understanding of the pathophysiological mechanism of cardiovascular disease in menopausal women and the clinical application of EXD in the prevention and treatment of cardiovascular disease in menopausal women. In addition, this study also provides preliminary work for further exploring the molecular mechanism of abnormal evolution of cardiac function and glucose and lipid metabolism in OVX rats treated with EXD.

## Data Availability

The data that support the findings of this study are available from the corresponding author.

## Additional Points

*Concoction Method.* The method for preparing *Morinda officinalis Radix* (Bajitian) and Epimedii Folium (Yinyanghuo) was as follows: *Morinda officinalis Radix* (Bajitian) was cooked at 90∼100°C for 80∼100 minutes. Then, it was cooked in the same pot as licorice soup until licorice soup ran out. Next, it was cut into sections and dried. The method for making licorice soup was as follows: water was added to licorice tablets at 10× by volume. This was fried 2 times, with the first occurring for 1 hour and the second occurring for 30 minutes. Then, the two fried licorice decoctions were combined. 0.06 kg of licorice was used for each 1 kg of *Morinda officinalis Radix* (Bajitian). For *Epimedii Folium* (Yinyanghuo), suet oil was heated until it melted. Then, *Epimedii Folium* (Yinyanghuo) was added and stirred. The temperature was set to 200°C, and the simmering powder temperature (80∼120°C) was used. The samples were fried for 10 minutes until the surface was even and shiny. When it turned yellow-green, it was removed and allowed to cool. For every 1 kg of net *Epimedii Folium* (Yinyanghuo), 0.2 kg of suet oil was used.

## Supplementary Materials

Supplementary Materials Concoction Method. The method for preparing *Morinda officinalis Radix* (Bajitian) and Epimedii Folium (Yinyanghuo) is as follows. *Morinda officinalis Radix* (Bajitian) was cooked at 90∼100°C for 80∼100 minutes. Then, it was cooked in the same pot as the licorice soup until the licorice soup ran out. Next, it was cut into sections and dried. The method for making licorice soup was as follows: water was added to licorice tablets at 10× by volume. This was fried 2 times, with the first occurring for 1 hour and the second occurring for 30 minutes. Then, the two fried licorice decoctions were combined. 0.06 kg of licorice was used for each 1 kg of *Morinda officinalis Radix* (Bajitian). For *Epimedii Folium* (Yinyanghuo), suet oil was heated until it melted. Then, the *Epimedii Folium* (Yinyanghuo) was added and stirred. The temperature was set to 200°C, and the simmering powder temperature (80∼120°C) was used. The samples were fried for 10 minutes until the surface was even and shiny. When it turned yellow‒green, it was removed and allowed to cool. For every 1 kg of net *Epimedii Folium* (Yinyanghuo), 0.2 kg of suet oil was used.
