# Supplementary material for: Dynamic Evolution of Cardiac Function and Glucose and Lipid Metabolism in Ovariectomized Rats and the Intervention Effect of Erxian Decoction
Source: Evid Based Complement Alternat Med. 2022 Dec 17;2022:8090868. doi: 10.1155/2022/8090868 (PMC9789914; doi:10.1155/2022/8090868)
Supplement: Supplementary Materials — Concoction Method. The method for preparing Morinda officinalis Radix (Bajitian) and Epimedii Folium (Yinyanghuo) is as follows. Morinda officinalis Radix (Bajitian) was cooked at 90∼100°C for 80∼100 minutes. Then, it was cooked in the same pot as the licorice soup until the licorice soup ran out. Next, it was cut into sections and dried. The method for making licorice soup was as follows: water was added to licorice tablets at 10× by volume. This was fried 2 times, with the first occurring for 1 hour and the second occurring for 30 minutes. Then, the two fried licorice decoctions were combined. 0.06 kg of licorice was used for each 1 kg of Morinda officinalis Radix (Bajitian). For Epimedii Folium (Yinyanghuo), suet oil was heated until it melted. Then, the Epimedii Folium (Yinyanghuo) was added and stirred. The temperature was set to 200°C, and the simmering powder temperature (80∼120°C) was used. The samples were fried for 10 minutes until the surface was even and shiny. When it turned yellow‒green, it was removed and allowed to cool. For every 1 kg of net Epimedii Folium (Yinyanghuo), 0.2 kg of suet oil was used. [file 8090868.f1.docx]

**Concoction Method**

The method for preparing *Morinda officinalis Radix*(Bajitian) and *Epimedii Folium*(Yinyanghuo) is as follows.

*Morinda officinalis Radix*(Bajitian) was cooked at 90~100 °C for 80~100 minutes. Then, it was cooked in the same pot as the licorice soup until the licorice soup ran out. Next, it was cut into sections and dried. The method for making licorice soup was as follows: water was added to licorice tablets at 10× by volume. This was fried 2 times, with the first occurring for 1 hour and the second occurring for 30 minutes. Then, the two fried licorice decoctions were combined. 0.06 kg of licorice was used for each 1 kg of *Morinda officinalis Radix*(Bajitian).

For *Epimedii Folium*(Yinyanghuo), suet oil was heated until it melted. Then, the *Epimedii Folium*(Yinyanghuo) was added and stirred. The temperature was set to 200 °C, and the simmering powder temperature (80~120 °C) was used. The samples were fried for 10 minutes until the surface was even and shiny. When it turned yellow‒green, it was removed and allowed to cool. For every 1 kg of net *Epimedii Folium*(Yinyanghuo), 0.2 kg of suet oil was used.
